# Effect of Inoculating Two Bacteriocin-Producing *Lactiplantibacillus plantarum* Strains at Ensiling on In Vitro Rumen Fermentation and Methane Emissions of Alfalfa Silage with Two Dry Matter Contents

**DOI:** 10.3390/ani13030384

**Published:** 2023-01-23

**Authors:** Ziqian Li, Fuhou Li, Zohreh Akhavan Kharazian, Xusheng Guo

**Affiliations:** 1 State Key Laboratory of Grassland Agro-Ecosystems, School of Life Sciences, Lanzhou University, Lanzhou 730013, China; 2 Probiotics and Biological Feed Research Center, Lanzhou University, Lanzhou 730013, China

**Keywords:** *Lactiplantibacillus plantarum*, bacteriocin, in vitro rumen, methane emissions, digestibility

## Abstract

**Simple Summary:**

Developing alternative antibiotics is crucial for sustainable animal production worldwide. Bacteriocin has been considered a potential alternative to antibiotics due to its lack of drug resistance, lack of residues, and environmental friendliness. In this study, we investigated the effects of inoculating two bacteriocin-producing strains, *Lactiplantibacillus plantarum* ATCC14917 and LP1-4, at ensiling on the in vitro ruminal fermentation characteristics and methane emissions of alfalfa silage with two different dry matter contents (355 g/kg fresh weight, moderate dry-matter content; 428 g/kg fresh weight, high dry-matter content). The results showed that inoculating with ATCC14917 and LP1-4 at ensiling reduced in vitro rumen methane production and enhanced the dry matter digestibility of ensiled alfalfa. Therefore, bacteriocin-producing *L. plantarum* ATCC14917 and LP1-4 inoculants can potentially mitigate ruminal methane emissions but without an adverse effect on the rumen fermentation of the inoculated alfalfa silage.

**Abstract:**

The objective of this study was to investigate the effects of inoculating two bacteriocin-producing strains, *Lactiplantibacillus plantarum* ATCC14917 and LP1-4, at ensiling on the in vitro ruminal fermentation characteristics and methane production of alfalfa silage with two dry matter (DM)contents. Before ensiling, fresh alfalfa was wilted to a moderate DM content (355 g/kg) and a high DM content (428 g/kg). The wilted alfalfa was treated with (1) distilled water (control), (2) commercial strain *L. plantarum* MTD/1 (MTD/1), (3) bacteriocin-producing *L. plantarum* ATCC14917 (ATCC14917), and (4) a bacteriocin-like substance producing *L. plantarum* LP1-4 (LP1-4) at 1 × 10^5^ colony forming units (CFU)/g fresh weight. After 90 d of ensiling, the silages were used for in vitro rumen fermentation. Inoculation with the two bacteriocin-producing strains at ensiling remarkably reduced (*p* < 0.05) in vitro ruminal CH_4_ production and enhanced DM digestibility compared with the control group regardless of DM content. For silages with high DM content, inoculation with the bacteriocin-producing strains even increased (*p* < 0.05) in vitro ruminal total volatile fatty acid production. Therefore, the bacteriocin-producing inoculants have a great potential to mitigate ruminal methane emission but without an adverse effect on rumen fermentation of the inoculated alfalfa silage.

## 1. Introduction

It is known that the greenhouse effect of methane (CH_4_) is nearly 25 times that of carbon dioxide (CO_2_) [1]. Ruminant CH_4_ emissions account for 16% of global greenhouse gases and 33% of global anthropogenic CH_4_ emissions [2]. As reported in a previous study, 75 million tons (Tg) of CH_4_ was discharged from the gut of cattle around the world in 2004, and about 9 Tg of CH_4_ was discharged from small ruminants such as sheep and goats [3]. In addition, about 87% of CH_4_ in ruminants originates from the rumen and 13% from the hindgut [4,5]. Therefore, how to reduce CH_4_ emissions from ruminants without affecting feed digestion is always the focus worldwide.

Antibiotics have been shown to reduce ruminal CH_4_ emissions, but antibiotic residues in animal products and the emergence of antibiotic-resistance genes in the environment have led to the worldwide prohibition of antibiotics in animal husbandry [6,7,8]. Bacteriocins are antimicrobial peptides produced by bacteria and can accelerate the death of homologous bacteria by disrupting the lipid II metabolism of bacterial cell membranes or inhibiting the replication of nucleic acids [9,10]. They have been considered a potential alternative to antibiotics due to their lack of drug resistance, lack of residues, and environmental friendliness. Many lactic acid bacteria (LAB) produce bacteriocins. Some researchers have reported that adding bacteriocin alone in rumen culture or inoculating LAB without bacteriocin-producing ability in silage has obvious effects on reducing ruminant CH_4_ emissions [6,11]. Thus, the authors hypothesized that inoculating bacteriocin-producing LAB in silage probably exhibits the same role as the addition of bacteriocin alone in reducing in vitro ruminal methane emissions and improving the silage quality. However, few studies have investigated the effect of bacteriocin-producing LAB on silage fermentation and subsequent rumen digestion and CH_4_ emission.

Thus, this study investigated the impact of two bacteriocin-producing *Lactiplantibacillus plantarum* inoculants on in vitro ruminal digestibility, fermentation characteristics, and methane production of alfalfa silage with two dry matter (DM) contents. This provided a theoretical basis for the application of bacteriocin-producing *L. plantarum* instead of antibiotics in reducing the CH_4_ emission of ruminants.

## 2. Materials and Methods

### 2.1. Strain Preparation

The inoculants used in the present study included (1) *L. plantarum* MTD/1 (NCIMB 40027), a non-bacteriocin-producing silage inoculant, purchased from Ecosyl Products Ltd., Stokesley, UK; (2) *L. plantarum* ATCC14917, a class IIa bacteriocin-producing strain that was purchased from American Type Culture Collection [12]; (3) *L. plantarum* LP1-4, a bacteriocin-like-substance-producing strain, isolated and screened from turbot intestine originally sampled in Jinzhou, China [13]. The three strains were activated twice with a 1% (*v*/*v*) inoculation rate in MRS broth and cultured at 37 °C for 18 h before silage making.

### 2.2. Alfalfa Silage Preparation

Alfalfa (*Medicago sativa.*) was cultivated and manually harvested from four experimental fields in Dingxi (N35°58′, E104°62′), Gansu province, China, on July 2022. The harvested alfalfa from each field was divided into two portions and naturally wilted to DM contents of 355 (moderate DM content) and 428 (high DM content) g/kg fresh weight (FW), respectively. The water-soluble carbohydrates, crude protein, neutral detergent fiber (aNDF; using heat-stable α-amylase), and acid detergent fiber (ADF) contents of moderate and high DM were 31.5 vs. 36.7, 177 vs. 172, 366 vs. 391, and 283 vs. 294 g/kg DM, respectively. The wilted forages from four harvested fields at each DM content served as four experimental replicates. After that, the forages were chopped into small pieces, roughly 1−2 cm in length. The forage from each of the four fields was then treated with the following treatments: (1) distilled water (control), (2) *L. plantarum* MTD/1 (MTD/1), (3) *L. plantarum* ATCC14917 (ATCC 14917), and (4) *L. plantarum* LP1-4 (LP1-4) at 1 × 10^5^ colony forming units (CFU)/g FW. Before the experiment, each LAB culture was centrifuged at 8000× *g* for 5 min after 24 h of cultivation, and the precipitation was suspended in sterile water to achieve an application rate of 2 × 10^7^ CFU of viable cells/mL and subsequently evenly sprayed onto the chopped alfalfa at 5 mL/500 g FW. An equal volume of sterile water was used to treat the control. Each treated pile (approximately 500 g) was single-sealed in a plastic vacuum bag (density 0.91 to 0.93 g/cm^3^; vacuum degree 0.1 Mpa) and fermented for 90 d.

### 2.3. Lactic Acid Fermentation Characteristics and Fiber Analysis

On the designated day, a 20 g sample (including fresh samples and silages) was taken from each silo randomly, soaked with 180 mL sterile water for 24 h at 4 °C, and filtered with four layers of sterile gauze. The pH of the silage filtrate was instantly determined with a pH meter (PHSJ-3F, CANY, Shanghai, China). Lactic acid (LA) was measured according to the method of Yang et al. [14]. Approximately 20 g of fresh and silage samples were oven-dried at 65 °C for 72 h to determine DM, aNDF, and ADF contents using the methods described by Van Soest et al. [15].

### 2.4. In Vitro Rumen Fermentation

After 90 d of fermentation, the silages with two DM contents were subject to in vitro ruminal fermentation trials. Three rumen-fistulated Hu sheep (60 ± 5 kg) were used as rumen fluid donors for in vitro culture. The sheep were rationed twice a day (08:00 and 18:00) with a total mixed ration (TMR) pellet containing 58% corn, 19% wheat bran, 18% soybean meal, 1% baking soda, and 4% vitamin and mineral supplement. The collected rumen fluid was filtered using four layers of sterile gauze, equal volume mixed and poured into sterilized bottles (1500 mL) preheated at 39 °C. CO_2_ was immediately infused into the sterilized bottle filled with filtrate to eliminate air, and the filtrate was transported to the laboratory within 20 min. The artificial buffer solution was prepared according to Menke and Steingass [16], with each 1 L artificial buffer solution (pH 7.0) containing 237 mL buffer solution (4.0 g/L NH_4_HCO_3_ +35.0 g/L NaHCO_3_), 237 mL macro element solution (5.7 g/L Na2HPO_4_ + 6.2 g/L KH_2_PO_4_), 0.12 mL trace element solution (13.2 g/100 mL CaCl_2_∙2H_2_O + 10.0 g/100 mL MnCl_2_∙2H_2_O + 1.00 g/100 mL CoCl_2_∙6H_2_O + 8.00 g/100 mL FeCl_3_ + 6H_2_O), 1.22 mL resazurin solution (100 mg/100 mL), 50 mL reducing agent solution (285 mg/50 mL Na_2_S∙7H_2_O + 800 mg/50 mL NaOH), and 474 mL distilled water [16]. Before blending with filtrate in a ratio of approximately 4:1 (*v*/*v*), the artificial buffer was preheated to 39 °C and continuously infused with CO_2_. The crushed silage samples from each silo were prepared in quadruplicate (a total of 128 subsamples, 16 replicates by a treatment). A 0.5 g sample was weighed and put into a fiber bag (F57, Ankom Technology, New York, NY, USA) and heat-sealed. Another three bags were used as a blank. All bags were pre-dried to constant weight and put into 100 mL sterile glass sealed bottles. Half of the bottles (two bottles in each silage replicate) were linked with a microbial fermentation gas production automatic recorder (Boxiang Xingwang Technology Co., Ltd., Beijing, China) and filled with 90 mL of blended in vitro fermentation solution (20 mL filtrate and 70 mL artificial buffer solution) to monitor total gas production. The remaining two bottles were linked with gas-collecting bags (500 mL) for subsequent CH_4_ analysis. All bottles were cultured for 48 h at 39 °C.

After incubation, the fiber bags were washed with warm water until colorless and dried at 105 °C for 3 h before calculating the in vitro DM digestibility (IVDMD). Subsequently, 0.2 mL of 25% H_3_PO_4_ containing 2-methyl butyric acid was added to 1 mL of incubated fluid for volatile fatty acid (VFA) analysis using gas chromatography (trace 1300, Thermo Fisher Scientific Inc., Singapore) with an electrical conductivity detector and capillary column (30 m × 0.32 × mm 0.50 × µm; Lan-zhou Zhongke Kaidi Chemical New Technology Co., Ltd., China) according to the method described by Chen et al. [17]. The ammonia (NH_3_) concentration in the incubated fluid was determined according to the method of Broderick and Kang [18]. The gas collected by the gas collecting bags was used for the determination of CH_4_ content using gas chromatography with a flame ionization detector and 19095P-QO3 column (30 m × 0.53 mm × 40.00 µm; Agilent Technologies Inc., Santa Clara, CA, USA) according to the method of Chen et al. [17].

### 2.5. Statistical Analysis

The data of silage LA fermentation, fiber content, and in vitro ruminal fermentation parameters were analyzed using the general linear model procedure of SPSS 20.0 (IBM Co., Armonk, NY, USA) according to 2 (DM) × 4 (treatment) experimental design with the fixed factors of treatment, DM content, and their interaction. DM’s effects within each treatment were analyzed using Turkey’s multiple comparisons when the interaction was significant at *p* < 0.05.

## 3. Results

### 3.1. Lactic Acid Fermentation Characteristics and Fiber Contents of Silages at Moderate and High DM Contents after 90 Days

**Figure 1:**
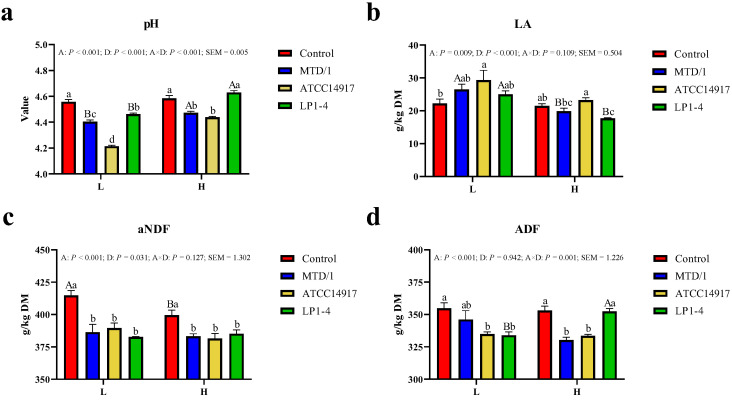
Lactic acid fermentation characteristics and fiber contents of alfalfa silages at moderate and high DM contents after 90 days. (**a**) PH, (**b**) LA, (**c**) aNDF, and (**d**) ADF. LA, lactic acid; aNDF, neutral detergent fiber analyzed with heat-stable α-amylase; ADF, acid detergent fiber; DM, dry matter. a–d: Different lowercase letters indicate significant differences among treatments in the same DM level (*p* < 0.05). A–B: Different capital letters indicate significant differences between DM levels in the same treatment (*p* < 0.05).

There were A × D interactions (*p* < 0.05) for pH and ADF (Figure 1a,d). All inoculants reduced (*p* < 0.05) silage pH compared with the control group, and ATCC14917 had the lowest (*p* < 0.05) pH value at moderate DM content, while MTD/1 and ATCC14917 with the high DM content had a lower (*p* < 0.05) pH value than the control group and LP1-4, respectively. Compared with control, ADF contents were lower (*p* < 0.05) in the two bacteriocin-producing LAB treatments at moderate DM level, while at high DM silage, ADF contents in MTD/1 and ATCC14917 groups were lower (*p* < 0.05) than that in the other groups. On average, LA concentration was higher (*p* < 0.05) in moderate DM silages versus high DM silages (25.8 vs. 20.6 g/kg DM), and ATCC14917 had the highest (*p* < 0.05) LA concentration regardless of silage DM level (Figure 1b). All inoculants decreased (*p* < 0.05) aNDF contents for both DM contents compared with the control groups (Figure 1c).

### 3.2. CH_4_ Production and Fermentation Characteristics of In Vitro Ruminal Fermentation of Alfalfa Silage

There were A × D interactions (*p* < 0.05) for total gas, CH_4_, CH_4_-to-total gas, in vitro IDVMD, total VFA, butyrate, and valerate (Table 1). Silages ensiled at moderate DM content increased (*p* < 0.05) CH_4_-to-total gas ratio, acetate-to-propionate ratio, IDVMD, CH_4_, total VFA, acetate, butyrate, isobutyrate, and isovalerate concentrations compared with the silages at high DM content regardless of treatments. All inoculants reduced (*p* < 0.05) CH_4_ concentrations compared with the control group at both DM contents; ATCC4917 with the moderate DM content and the two bacteriocin-producing LAB treatments with the high DM content had lower (*p* < 0.05) CH_4_ concentrations than MTD/1. The two bacteriocin-producing LAB decreased (*p* < 0.05) CH_4_-to-total gas ratio compared with the control group at moderate DM content, while a lower (*p* < 0.05) CH_4_-to-total gas ratio was observed in all inoculants at high DM content. The CH_4_-to-total gas ratio in ATCC4917 with moderate DM content and the two bacteriocin-producing LAB groups with high DM content was lower (*p* < 0.05) than that in MTD/1. IDVMD was increased (*p* < 0.05) in all inoculated silages with moderate DM content and the two bacteriocin-producing LAB treated-silages with high DM content. Acetate concentration and acetate to propionate ratio in the two bacteriocin-producing LAB treatments were lower (*p* < 0.05) than in the control group at both DM contents. LP1-4 with the moderate DM content and the two bacteriocin-producing LAB with the high DM content increased (*p* < 0.05) propionate concentrations compared with the control group. Butyrate concentrations in LP1-4 with the moderate DM content and the two bacteriocin-producing LAB treated-silages with the high DM content were lower (*p* < 0.05) than in the control group. Compared with the control group, inoculating the two bacteriocin-producing LAB in silage increased (*p* < 0.05) the ruminal isobutyrate and isovalerate concentrations only at high DM content. Valerate concentration in the two bacteriocin-producing LAB treated-silages was lower (*p* < 0.05) than that in the control group at moderate DM content, while an increased (*p* < 0.05) valerate concentration was observed in the two bacteriocin-producing LAB treatments at high DM content.

## 4. Discussion

The present study showed that alfalfa silages ensiled at high DM content increased pH values and decreased LA concentrations compared with the silages at moderate DM content regardless of treatments, consistent with previous studies [19,20,21]. Moreover, the lowest pH and the highest LA concentration in ATCC14914 regardless of the DM contents, showed that the silage fermentation effect of the ATCC14917 was the most desirable. In addition, the fiber contents in all inoculant treatments, regardless of DM contents, can be explained by the impact of pH because low pH can enhance the ability of plant cellulase to degrade structural carbohydrates during ensiling [22,23].

The in vitro ruminal fermentation trial showed that IDVMD, CH_4_, acetate, and butyrate concentrations with moderate DM content were higher than those with high DM content regardless of treatments, which contributed to the higher aNDF content in moderate DM content versus high DM content (393 vs. 387 g/kg DM) because high fiber content determined the increase in in vitro ruminal acetate concentration, accompanied by the increase in CH_4_ production [24]. Moreover, elevated acetate concentration at moderate DM content, regardless of treatments, may suggest an enhancement of cellulose bacteria’s role in acetate fermentation, thereby increasing digestibility [24]. As expected, ATCC14917 and LP1-4 reduced CH_4_ productions by 68.8% and 43.9% at moderate DM content, respectively, and they reduced CH_4_ productions by 63.6% and 78.6% at high DM content, respectively. This was consistent with the results of the application of LAB or nisin in vitro trials [6,11]. Moreover, the two bacteriocin-producing strains enhanced the forage DM digestibility at both DM contents. However, Shen et al. [6] reported that nisin decreased CH_4_ production without increasing digestibility, which was distinct from the result of inoculation with LAB. Oskoueian et al. [25] showed that *L. plantarum,* a silage inoculant, could diminish CH_4_ emissions and improve DM digestibility. Generally, a decrease in CH_4_ production is usually accompanied by a reduction in digestibility [24]. In previous studies, the inhibition of the ruminal fibrolytic bacteria such as *Ruminococcus flavefaciens* and *Ruminococcus albus* by nisin resulted in an inhibition of hydrogen forming through the acetyl-coA pathway, thereby reducing methane emission [26,27]. However, ATCC14917 in this study could produce a large amount of LA in addition to bacteriocins during ensiling, thereby probably enhancing the electron-accepting reaction in the rumen [28]. This allows the metabolic hydrogen produced by glucose during rumen fermentation to synthesize VFA (mainly propionate) instead of being utilized by methanogens to generate CH_4_ [29]. This can be confirmed by the higher in vitro ruminal propionate concentration, lower acetate and butyrate concentrations and acetate to propionate ratio in ATCC14917 treatment. Previous studies also confirmed that LA produced by *L. plantarum* during ensiling could promote the availability of hydrogen via the acrylate metabolic pathway to produce more propionate, which leads to a reduction in the acetate and butyrate metabolic pathway for CH_4_ production [30,31]. The increased isobutyrate and isovalerate concentrations in the two bacteriocin-producing LAB treatments at high DM content were consistent with the increased DM digestibility because the increase in branched-chain fatty acid (BCVFA) could enhance the digestion capacity of fibrobacteria, thereby improving DM digestibility [32]. Moreover, all inoculants and the two bacteriocin-producing strains decreased aNDF and ADF, respectively, which also improved the DM digestibility of in vitro fermentation [33]. In addition, the increased BCVFA concentrations in the two bacteriocin-producing LAB treatments at high DM content also imply enhanced deaminations of amino acids [24]. However, the NH_3_ concentration in the present study remained constant in the two treatments, and the NH_3_ concentration in the in vitro rumen only depends on the balance between the rate of formation and utilization of NH_3_ by microorganisms, suggesting that microbial protein synthesis was increased.

The bacteriocin concentration during silage fermentation was not determined in the present study. In future research, the qualitative or quantitative analysis of bacteriocin concentration in silages and rumen-incubated fluid will be checked by using high-performance liquid chromatography/mass spectrometry (HPLC-MS)

## 5. Conclusions

ATCC14917 and LP1-4 had better effects than MTD/1 in reducing in vitro rumen methane production and improving the DM digestibility of alfalfa silage. Therefore, the bacteriocin-producing *L. plantarum* ATCC14917 and LP1-4 inoculants have a great potential to be used as silage additives to improve silage fermentation quality and mitigate methane emissions but without an adverse effect on rumen fermentation of the inoculated silage.

## Figures and Tables

**Table 1:** In vitro ruminal fermentation characteristics of silage inoculated with bacterial strains.

Iterms ^1^	M					H						Effects ^3^		
	Control	MTD/1	ATCC-14917	LP1-4	Mean	Control	MTD/1	ATCC-14917	LP1-4	Mean	SEM ^2^	A	D	A × D
pH	6.67	6.67	6.74	6.75	6.71	6.69	6.68	6.72	6.70	6.70	0.014	0.359	0.682	0.801
Total gas (mL/g DM)	103 ^Ba^	86.6 ^Bb^	83.5 ^b^	99.0 ^a^	93.0	124 ^Aa^	106 ^Ab^	90.5 ^c^	92.5 ^c^	103	18.473	<0.001	<0.001	<0.001
CH_4_ (mL/g DM)	11.3 ^a^	7.34 ^b^	3.53 ^c^	6.34 ^Abc^	7.14	9.88 ^a^	7.23 ^b^	3.60 ^c^	2.11 ^Bc^	5.71	0.977	<0.001	0.003	0.006
CH_4_-to-total gas (% mL)	11.0 ^a^	8.49 ^Aab^	4.27 ^c^	7.29 ^Abc^	7.77	8.00 ^a^	6.85 ^Bb^	3.92 ^c^	2.29 ^Bc^	5.27	1.151	<0.001	<0.001	0.011
IDVMD (g/kg DM)	65.5 ^Ab^	70.3 ^Aa^	71.7 ^Aa^	69.3 ^Aa^	69.2	52.1 ^Bb^	54.5 ^Bb^	63.4 ^Ba^	61.5 ^Ba^	57.9	2.609	<0.001	<0.001	0.001
NH_3_ (mg/100 mL)	22.9	24.3	24.8	25.1	24.2	23.2	23.1	27.5	26.2	25.0	2.344	0.006	0.230	0.277
Total VFA (mM)	72.0 ^A^	68.6 ^A^	67.8 ^A^	68.0 ^A^	69.1	54.2 ^Bab^	50.4 ^Bb^	57.0 ^Ba^	57.5 ^Ba^	54.8	4.409	0.033	<0.001	0.006
Acetate (mM)	40.7 ^Aa^	37.5 ^Aab^	36.9 ^Ab^	36.4 ^Ab^	37.9	30.8 ^Ba^	29.2 ^Bb^	28.7 ^Bb^	29.2 ^Bb^	29.5	1.032	<0.001	<0.001	0.171
Propionate (mM)	10.3 ^b^	10.7 ^b^	12.1 ^ab^	13.2 ^a^	11.6	11.0 ^b^	11.0 ^b^	14.9 ^a^	14.6 ^a^	12.9	1.060	<0.001	0.007	0.233
Acetate to propionate	3.98 ^Aa^	3.50 ^Aab^	3.05 ^Abc^	2.76 ^Ac^	3.32	2.78 ^Ba^	2.54 ^Bab^	2.13 ^Bbc^	1.80 ^Bc^	2.32	0.055	<0.001	<0.001	0.703
Butyrate (mM)	15.1 ^Aa^	12.8 ^Aab^	12.3 ^Aab^	11.3 ^Ac^	12.9	8.52 ^Ba^	5.76 ^Bc^	7.37 ^Bb^	7.38 ^Bb^	7.26	0.958	0.001	<0.001	0.039
Isobutyrate (mM)	1.20	1.18 ^A^	1.29 ^A^	1.44	1.28	0.66 ^b^	0.61 ^Bb^	1.04 ^Ba^	1.14 ^a^	0.86	0.062	0.041	0.001	0.577
Valerate (mM)	2.30 ^a^	2.01 ^Bab^	1.77 ^Bb^	1.84 ^Bb^	1.98	2.00 ^b^	2.62 ^Aab^	3.16 ^Aa^	3.23 ^Aa^	2.75	0.045	0.027	<0.001	<0.001
Isovalerate (mM)	2.45	3.30 ^A^	3.37 ^A^	3.90 ^A^	3.26	1.15 ^b^	1.19 ^Bb^	1.86 ^Ba^	1.90 ^Ba^	1.52	0.266	0.012	<0.001	0.486

^1^ CH_4_, methane; IDVMD, in vitro dry matter digestibility; NH_3_, ammonia; VFA, volatile fatty acids. ^2^ SEM, standard error of the means. ^a–c^ Different lowercase letters indicate significant treatment differences (*p* < 0.05). ^A,B^ Different uppercase letters indicate significant differences in dry matters (*p* < 0.05). ^3^ A, additive; D, dry matter; A × D, the interaction between additive and dry matter; M, moderate dry matter content; H, high dry matter content.

## Data Availability

The data presented in this study are available upon request from the corresponding author.
